# Comparing Ictal Cardiac Autonomic Changes in Patients with Frontal Lobe Epilepsy and Temporal Lobe Epilepsy by Ultra-Short-Term Heart Rate Variability Analysis

**DOI:** 10.3390/medicina57070666

**Published:** 2021-06-28

**Authors:** Sung-Min You, Hyun-Jin Jo, Baek-Hwan Cho, Joo-Yeon Song, Dong-Yeop Kim, Yoon-Ha Hwang, Young-Min Shon, Dae-Won Seo, In-Young Kim

**Affiliations:** 1 Department of Biomedical Engineering, Hanyang University, 222, Wangsimni-ro, Seongdong-gu, Seoul 04763, Korea; seungmin@hanyang.ac.kr; 2 Department of Neurology, Samsung Medical Center, Sungkyunkwan University School of Medicine, 81, Irwon-ro, Gangnam-gu, Seoul 06351, Korea; hyunjin530.jo@samsung.com (H.-J.J.); jooyeon.song@samsung.com (J.-Y.S.); dy2028.kim@samsung.com (D.-Y.K.); dbsgk83@naver.com (Y.-H.H.); sonogung@gmail.com (Y.-M.S.); 3 Medical AI Research Center, Samsung Medical Center, Sungkyunkwan University School of Medicine, 81, Irwon-ro, Gangnam-gu, Seoul 06351, Korea; baekhwan.cho@samsung.com; 4 Department of Medical Device Management and Research, Samsung Advanced Institute for Health Sciences & Technology, Sungkyunkwan University School of Medicine, 81, Irwon-ro, Gangnam-gu, Seoul 06351, Korea

**Keywords:** epilepsy, cardiac autonomic alteration, heart rate variability, frontal lobe epilepsy, temporal lobe epilepsy

## Abstract

*Background and Objectives*: Abnormal epileptic discharges in the brain can affect the central brain regions that regulate autonomic activity and produce cardiac symptoms, either at onset or during propagation of a seizure. These autonomic alterations are related to cardiorespiratory disturbances, such as sudden unexpected death in epilepsy. This study aims to investigate the differences in cardiac autonomic function between patients with temporal lobe epilepsy (TLE) and frontal lobe epilepsy (FLE) using ultra-short-term heart rate variability (HRV) analysis around seizures. *Materials and Methods*: We analyzed electrocardiogram (ECG) data recorded during 309 seizures in 58 patients with epilepsy. Twelve patients with FLE and 46 patients with TLE were included in this study. We extracted the HRV parameters from the ECG signal before, during and after the ictal interval with ultra-short-term HRV analysis. We statistically compared the HRV parameters using an independent t-test in each interval to compare the differences between groups, and repeated measures analysis of variance was used to test the group differences in longitudinal changes in the HRV parameters. We performed the Tukey–Kramer multiple comparisons procedure as the post hoc test. *Results*: Among the HRV parameters, the mean interval between heartbeats (RRi), normalized low-frequency band power (LF) and LF/HF ratio were statistically different between the interval and epilepsy types in the t-test. Repeated measures ANOVA showed that the mean RRi and RMSSD were significantly different by epilepsy type, and the normalized LF and LF/HF ratio significantly interacted with the epilepsy type and interval. *Conclusions*: During the pre-ictal interval, TLE patients showed an elevation in sympathetic activity, while the FLE patients showed an apparent increase and decrease in sympathetic activity when entering and ending the ictal period, respectively. The TLE patients showed a maintained elevation of sympathetic and vagal activity in the pos-ictal interval. These differences in autonomic cardiac characteristics between FLE and TLE might be relevant to the ictal symptoms which eventually result in SUDEP.

## 1. Introduction

Epilepsy is the second-most functionally distressing neurological disease worldwide and affects 6.38 out of 1000 people [1,2]. The most common types of epilepsy are temporal lobe epilepsy (TLE) and frontal lobe epilepsy (FLE). They are classified depending on the location of the epileptogenic zone, which can be differentiated using electrophysiological, semiological, and imaging findings [3,4,5]. TLE is generally characterized by a prolonged duration of automotor seizures and post-ictal confusion, while FLE patients more frequently tend to have hypermotor seizures with a shorter latency to secondary generalization [4,6]. Cardiovascular autonomic regulation is impaired in patients with TLE, FLE or chronic refractory epilepsy during the interictal state, manifested by impaired heart rate variability in response to various stimuli or the reduced uptake of 123I-metaiodobenzylguanidine, a marker of postganglionic sympathetic dysfunction [7,8,9,10].

Although the mechanism underlying autonomic dysfunction in epilepsy patients is not fully elucidated, alteration in cardiac ion channel expression and the detrimental effects of cortical epileptic discharge on centrally mediated cardiac outputs are the suggested pathophysiology [11]. Abnormal epileptic discharges in the brain also can affect the central brain regions that regulate autonomic activity and cause these cardiac symptoms, either at onset or during propagation of a seizure [12]. These autonomic alterations that are related to cardiorespiratory disturbances, such as tachycardia, bradycardia or asystole, can result in sudden unexpected death in epilepsy (SUDEP) [12,13,14,15,16]. Among the cardiorespiratory disturbances, ictal tachycardia is most commonly reported [17]. On the other hand, ictal bradycardia is a relatively rare phenomenon that can be identified in less than 6% of complex partial seizures [18]. Ictal asystole is a more infrequent symptom that was observed in 0.27% of 6825 patients who underwent video-EEG monitoring [19]. Excessive activation of the sympathetic nervous system due to intense seizures possibly contributes to cardiopulmonary dysfunction [20,21] and prolonged suppression of brain activity [22], which can cause impaired arousal independent of the functions of the heart. These intense seizures that are characteristic of epilepsy can cause compensative responses (e.g., elevated adenosine levels) that can eventually result in SUDEP [23].

Heart rate variability (HRV) analysis is considered one of the most accurate indicators of sympathovagal balance in the autonomic nervous system (ANS) [24]. Recently, there has been increased interest in monitoring HRV in epileptic patients [25] due to the association between dysregulation of the cardiac autonomic nervous system and long-term morbidity and mortality in epileptic patients [26,27]. HRV monitoring can be utilized to understand this pathophysiology and develop a preventive measure for SUDEP. Previous studies comparing HRV between temporal lobe epilepsy and frontal lobe epilepsy are scant, except for our previous report, in which only analysis of the heart rate was performed [28]. Another previous study compared HRV between frontal lobe epilepsy and controls, but their analysis was limited to the interictal state [10].

Conventional HRV analysis requires a long-term heart rate sequence (e.g., 24-h measurements). Long-term HRV is useful to assess the homeostatic mechanisms related to circadian rhythms, the core body temperature, sleep cycle and general metabolism. Therefore, long-term HRV has been considered the gold standard for clinical HRV assessment [29]. For example, 24-h measurements of HRV were used to predict cardiac risks such as acute myocardial infarction or chronic heart failure [30,31,32]. However, the applicability and reliability of short-term HRV analysis, which can be performed with shorter heart rate sequences (~5 min), have been established in various fields since 1996 [33,34,35]. Short-term HRV analysis is useful for focusing on the effects of the interaction between sympathetic and parasympathetic branches of the ANS and respiration sinus arrhythmia (RSA) rather than the effect of general circadian periodicity [36]. Short-term HRV analysis also can be used in epileptic patients to investigate cardiac alterations around ictal onsets [37,38,39,40].

Recently, several investigations have shown the possibility for HRV analysis with a shorter recording interval. This approach is called ultra-short-term HRV analysis, which refers to HRV analysis with less than five-minute intervals. Several researchers have investigated the reliability of this method. For example, Salahuddin et al. [41] showed that 60 s is enough to estimate the HRV parameters in 24 healthy subjects. Nussinovitch et al. [42,43] investigated the minimum length of heat rate sequences to estimate the HRV parameters computed within a five-minute interval. They showed that a 60-s interval is enough time to approximate most HRV parameters. Other studies [44,45,46] also demonstrated that ultra-short-term HRV analysis might be an effective surrogate method.

We hypothesized that ultra-short-term HRV analysis could be useful to assess the autonomic alterations around the ictal interval, which are related to the differences in the post-ictal conditions between epilepsy types. The objective of this study was to assess the differences in autonomic patterns within each interval (pre-ictal, ictal and post-ictal) between patients with FLE and patients with TLE using ultra-short-term HRV analyses.

## 2. Materials and Methods

### 2.1. Study Population

We retrospectively reviewed the medical records of the Samsung Medical Center Epilepsy Monitoring Unit between 2014 and 2019. Subjects were identified from a database with the following criteria: (1) definite TLE or FLE with electrophysiologic, semiologic and imaging findings, (2) the presence of ictal tachycardia, defined by HR > 100 bpm or a >20% increase in the baseline HR, (3) identifiable QRS complexes on an ictal ECG, (4) partial seizure without secondary generalization and (5) more than 180-s intervals between ictal events. Patients who had probable psychological paroxysmal events were excluded from this study.

This study included 58 patients (36 males and 22 females) between 8 and 71 years of age (mean age: 33.1 ± 15.7). Among them, 12 patients had frontal lobe epilepsy and 46 patients had temporal lobe epilepsy. The group of patients with frontal lobe epilepsy consisted of 3 females and 9 males with an age range of 8–70 years (mean age: 18.3 ± 7.8 years). The mean number of seizures for each patient with frontal lobe epilepsy was 5.8 (±3.4), and the mean ictal duration was 79.4 (±59.9) s. The group of patients with temporal lobe epilepsy consisted of 19 females and 27 males between 18 and 71 years of age (mean age: 36.5 ± 14.4 years). The mean number of seizures for each patient with temporal lobe epilepsy was 5.2 (±3.2), and the mean ictal duration was 98.8 (±50.4) s.

All patients underwent presurgical noninvasive evaluations, which included a video-EEG recording of habitual seizures, 3.0-Tesla brain magnetic resonance images (MRIs), 18F-FDG fluorodeoxyglucose (918F-FDG) positron emission tomography (PET) scans, subtracted 99m-Tc ECD ictal single-photon emission computed tomography scans, a complete neuropsychological examination and intracarotid amobarbital tests. For the presurgical evaluation, the patients were hospitalized in the epilepsy monitoring units to monitor their semiology and biosignals, such as by EEG and ECG. After these studies were reviewed in multidisciplinary intensive epilepsy conferences, the type of epilepsy and therapeutic strategies were identified.

This study was approved by the Institutional Review Board of Samsung Medical Center (IRB No. 2019-12-104-003, 8 January 2021) and conducted under the principles of the Declaration of Helsinki. The requirement for individual informed consent for this study was waived by the Institutional Review Board, as it was a retrospective study using electronic medical records.

### 2.2. Materials

According to the inclusion criteria, we retrospectively collected the electrocardiogram recordings of 309 seizure events (70 for FLE and 239 for TLE) from 58 patients (12 for FLE and 46 for TLE). The ECG recordings were conducted in the epilepsy monitoring unit. During hospitalization in the epilepsy monitoring unit, patients stopped taking antiepileptic drugs to inspect the semiology during seizures. The electroclinical data were reviewed separately by two epileptologists, who determined and marked the onsets and ends of seizures based on a scalp EEG to define the ictal interval. The EEG signals which could provide the most exact timing of a seizure were used to precisely define the onset and offset of each seizure. Along with the annotation from the EEG signals, we analyzed the ECG signals from three intervals. The first interval was the pre-ictal period, which was defined as the three minutes before seizure onset. The second interval was the post-ictal period, defined as the three minutes after seizure offset. Lastly, the ictal interval was the period during the seizure. The demographic and clinical data of the subjects are described in Table 1.

ECG data were collected using the NicoletOne LTM system (Natus Medical Incorporated, Pleasanton, CA, USA) with a sampling rate of 512 Hz. The ECGs were recorded with two electrodes located at the shoulders to record the lead 1 channel. After the ECG signals were acquired, we filtered them to remove noise due to subject movements, breathing and muscle electrical activity. We applied a third-order Butterworth 0.5 Hz high-pass filter to remove baseline wander and a 60-Hz notch filter to remove powerline interference [47]. A moving average low-pass filter with a 20-Hz cutoff frequency was used to filter electromyogram artifacts from the ECG signals [48].

With the filtered ECG signal, we extracted the R peak and the RR interval with the wavelet transform-based QRS complex detector method [49]. Ectopic beats were detected and excluded by a method based on the HRV signal [50]. A heartbeat was considered ectopic if there was a percentage change of 20% over the average five beats before and after. For ultra-short-term HRV analysis, a single ectopic beat could distort the result. Therefore, after the filtering process, every record was investigated by clinicians to confirm the reliability of the detected R points. The process of filtering and HRV analysis was conducted with MATLAB (The MathWorks, Inc., Natick, MA, USA) software.

### 2.3. Heart Rate Variability Analysis

The HRV parameters were extracted from the time and frequency domains [24]. For time domain analysis, we used the mean value of the RR interval (mean RRi), the standard deviation of all RR intervals (SDNN) and the root mean square of the successive differences (RMSSD). For frequency domain analysis, we applied the Lomb–Scargle (LS) periodogram to calculate the power spectral density of the HRV [51]. The LS periodogram is a spectral analysis method for unevenly sampled data. Previous research demonstrated that the LS periodogram is most appropriate for short-term spectral analysis than other spectral methods that require resampling. The LS periodogram has been shown to mitigate errors due to missing data, such as those due to corruption or ectopy [52,53]. Therefore, we used the LS periodogram to compute the frequency parameters of the ultra-short-term HRV analysis.

The power of the heart rate spectrum was divided into two components: low frequency (0.04–0.15 Hz) and high frequency (0.15–0.4 Hz). The power of each band was normalized by its ratio with the total power of the heart rate spectrum, except for the very low frequency band (less than 0.04 Hz). It is generally agreed that the LF band is influenced by both sympathetic and vagal activity, and the HF band represents only vagal activity [39,54,55]. To distinguish the influence of parasympathetic activity on the LF spectral power, the LF/HF ratio was calculated as an indicator of sympathovagal balance [56,57].

### 2.4. Statistical Analysis

Statistical analyses were performed with Stata, version 15 (StataCorp. 2017; Stata Statistical Software, Release 15; College Station, TX: StataCorp LLC). A *p*-value < 0.05 was considered statistically significant. First, we compared the HRV parameters in each interval to determine the differences between groups using the independent t-test for each ictal record. Then, repeated measures analysis of variance (ANOVA) was used to assay the group differences in the longitudinal HRV changes for the three intervals (pre-ictal, ictal and post-ictal) as the within-subjects factor and the group (FLE and TLE) as the between-subjects factor. As a post hoc test, we performed the Tukey–Kramer multiple comparisons test (α = 0.05) [58].

## 3. Results

The results of the independent t-test for each interval are described in Table 2. This shows that the mean RRi was statistically different by epilepsy type (*p* = 0.012 for pre-ictal and *p* < 0.001 for ictal and post-ictal). For the frequency domain HRV parameters, the normalized LF was statistically significantly different in the pre-ictal interval (*p* = 0.023) and ictal interval (*p* < 0.001) compared with the other groups. The LF/HF ratio was significantly distinct in the ictal interval (*p* = 0.010).

In Table 3, repeated measures ANOVA showed that the mean RRi and RMSSD were statistically different by epilepsy type (*p* < 0.001 for mean RRi and *p* = 0.011 for RMSSD). However, those two parameters did not significantly interact with the interval. On the other hand, the interaction between the epilepsy type and the interval was statistically significant for the normalized LF (*p* < 0.001) and LF/HF (*p* = 0.013).

Multiple post hoc comparisons showed that the FLE patients had significantly increased normalized LF for the ictal interval (M = 37.2, SD = 21.0) compared with that of the pre-ictal interval (M = 26.7, SD = 11.2) (Table S1). In addition, multiple linear regression analysis was performed, with the differences of the normalized LF between the pre-ictal, ictal and post-ictal intervals as the dependent variables (Table 4). Of the independent variables, the type of epilepsy and seizure frequency were significant predictors of the difference of the normalized LF between pre-ictal and ictal HRV. However, only the seizure frequency was a significant predictor of the difference in the normalized LF between ictal and post-ictal HRV.

**Figure 1:**
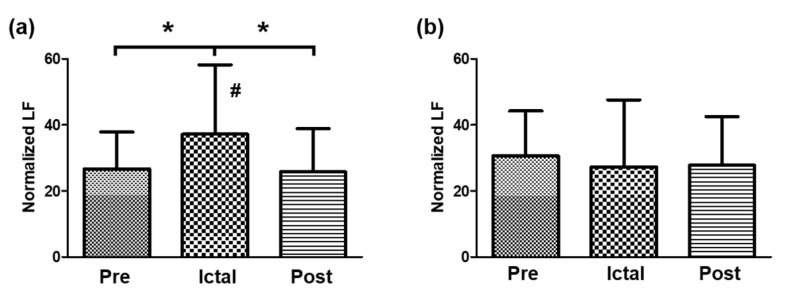
Comparison of normalized LF between intervals in the (**a**) FLE group and (**b**) TLE group. * Indicates significant different between intervals (pre-ictal, ictal, and post-ictal). # Indicates significant different between types (FLE and TLE) at same interval.

The normalized LF was reduced immediately in the post-ictal interval (M = 25.8, SD = 13.1) and showed a significant decrease from the ictal interval and no significant difference with that of the pre-ictal interval. On the other hand, TLE showed a modest decrease in the LF band from the pre-ictal interval to the post-ictal interval, but there were no significant differences in the normalized LF between intervals (Figure 1). 

**Figure 2:**
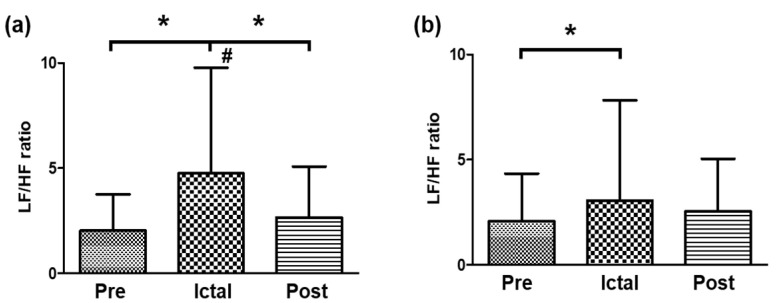
Comparison of the LF/HF ratio between intervals for the (**a**) FLE group and (**b**) TLE group. * Indicates significant different between intervals (pre-ictal, ictal, and post-ictal). # Indicates significant different between types (FLE and TLE) at same interval.

However, the LF/HF ratio showed interesting results. The FLE patients showed a significant increase in the LF/HF ratio for the ictal interval (M = 4.8, SD = 5.0) compared with that of the pre-ictal interval (M = 2.0, SD = 1.7) and decreased immediately in the post-ictal interval (M = 2.7, SD = 2.4). On the contrary, the TLE patients showed a significant increase in the ictal interval (M = 3.1, SD = 4.8) compared with the pre-ictal interval (M = 2.1, SD = 2.3), but the increase was prolonged in the post-ictal interval (M = 2.5, SD = 2.5) and showed no significant difference between the ictal and post-ictal intervals (Figure 2).

## 4. Discussion

This study aimed to investigate the cardiac autonomic differences between temporal lobe epilepsy and frontal lobe epilepsy by analyzing heart rate variability during the pre-ictal, ictal, and post-ictal intervals.

### 4.1. Alteration of ANS Control on HRV in the Ictal Interval

Most of the HRV parameters in the time and frequency domain changed significantly in the ictal interval (Table 2 and Table 3). In the time domain analysis, a decrease in the mean RRi was noted during the ictal interval, which manifested as ictal tachycardia. Increases in the standard deviation of all RR intervals (SDNN) and root mean square of the successive differences (RMSSD) were found during the ictal interval. In a previous report, the SDNN and RMSSD were increased in patients with epilepsy in the interictal condition compared with the normal controls [39]. This increase exhibited in epilepsy patients is thought to be augmented during the ictal period. The SDNN is related to the activity of both the sympathetic nervous system (SNS) and the parasympathetic nervous system (PNS), and it is highly correlated with the LF band power and total power [59]. The RMSSD reflects the beat-to-beat variance in HR and is primarily related to vagally mediated changes [29].

Among the frequency domain parameters, the observed combined increment of the LF/HF ratio supports the presence of higher sympathetic activity during the ictal interval. Many previous studies have reported those sympathetic dominance features in the interictal condition [10,25,39,40,60], but the trend is highlighted in the ictal interval.

### 4.2. Differences in HRV Parameters between Frontal Lobe Epilepsy and Temporal Lobe Epilepsy Patients

In a comparison of HRV parameters between frontal lobe epilepsy and temporal lobe epilepsy patients, we found that the TLE patients had a lower mean RRi during the pre-ictal, ictal and post-ictal periods compared with the FLE patients. Since the mean RRi is inversely correlated with the degree of tachycardia, the observed increases in the mean RRi in TLE patients indicate that ictal tachycardia was more prominent in this group, which is consistent with our previous report [28]. In detail, the pre-ictal period had a lower RRi, higher RMSSD and normalized LF in the TLE group. Since the RMSSD is used to estimate the vagal tone or parasympathetic activity, and the normalized LF is indicative of sympathetic and vagal activity, the observed findings in the TLE patients suggest that sympathetic and vagal activity are more active in the TLE group during the pre-ictal condition compared with FLE patients. Meanwhile, during the ictal interval, the normalized LF and LF/HF ratio were higher in the FLE group, indicating that the FLE patients had more activated sympathetic or vagal activity during the ictal interval. In the post-ictal period, however, there was no significant difference in the HRV parameters between the TLE and FLE patients.

### 4.3. Interval-Related Changes in HR Parameters

Both types of epilepsy are associated with an increase in HR during the ictal interval, which is related to ictal tachycardia, an autonomic manifestation of seizures [61,62]. Previous studies reported two patterns during the development of ictal tachycardia: one with a steady HR increase and the other with an abrupt HR increase [63,64]. The FLE patients in the ictal interval demonstrated a higher LF and LF/HF compared with both the pre-ictal and post-ictal intervals, indicating an abrupt sympathetic increase and decrease before and after the ictal period, respectively. In the TLE patients, on the other hand, a significant change was observed only in the LF/HF ratio from the pre-ictal to the ictal interval, indicating that the sympathetic tone increased in the ictal interval and remained high during the post-ictal period. This finding is in line with previous works [28,63] that demonstrated a steady increase in HR and slow recovery to baseline in TLE patients.

### 4.4. HRV Parameters and Their Clinical Interpretation

In this study, a pattern of HRV in the TLE patients was characterized by pre-ictal elevation of the sympathovagal tone and subsequent increase and maintenance in sympathetic activity during the ictal and post-ictal periods. On the contrary, the FLE patients were marked by an abrupt rise and fall of the sympathetic tone when entering and leaving the ictal period. An enhanced sympathovagal state in the pre-ictal TLE patients can be explained by anatomic resemblance or proximity of epileptogenic focus in the TLE to the adjacent limbic system, such as the amygdala, hippocampus or insula, which are known parts of the central autonomic network [65].

FLE patients, on the other hand, are characterized by a shorter seizure duration and more frequent secondary generalization than TLE patients, and these characteristics can be related to a more abrupt and stronger rise in the sympathetic tone during the ictal period in FLE patients. Since the thalamus is a subcortical structure known to be involved in ictal cardiac changes as well as in seizure generalization [15], it can be postulated that the thalamus is involved more frequently in FLE patients during the ictal event than it is in TLE patients.

In the temporal lobe, the amygdala is highly and widely connected with the autonomic structures of the hypothalamus and brainstem that can induce complex autonomic disturbances. Enhanced vagal activity in TLE patients during the ictal and post-ictal periods can be a compensatory mechanism against the prolonged elevation in the sympathetic tone observed in pre-ictal and post-ictal intervals. Meanwhile, the finding that an elevated sympathetic tone in the TLE patients remained high in the post-ictal period can be related to the phenomenon of prolonged post-ictal confusion in this group [4], because limbic activation is believed to be involved in both autonomic homeostasis and psychosomatic awareness [66]. With parasympathetic hyperactivity, which might be related to impaired autonomic regulation in the brainstem, post-ictal coactivation of sympathetic and parasympathetic systems increases the likelihood of SUDEP [67]. A meta-analysis of 39 studies that evaluated HRV in patients with epilepsy confirmed the hypothesis of sympathovagal imbalance in epilepsy, as shown by the lower values of parasympathetic activity when compared with the controls [68], and it was suggested that this might play a role in the mechanism of SUDEP. According to the adenosine hypothesis of SUDEP [69], seizure-induced adenosine release with impaired metabolic clearance by astrocytes, specifically in the brainstem, may trigger lethal apnea or cardiac arrest, which eventually results in SUDEP.

There are some limitations to this study. Although we ascertained the differences in HRV between TLE and FLE patients, the sample size was relatively small. Even though ictal tachycardia is the most common symptom among the cardiorespiratory disturbances in patients with epilepsy, further studies with other cardiorespiratory disturbances like ictal bradycardia and ictal asystole are needed for the understanding and prevention of SUDEP. Additionally, we focused on HRV patterns around the ictal interval with ultra-short-term HRV analysis. However, the HRV was also affected by the circadian rhythm and antiepileptic drugs. Therefore, a future study that considers the effects of a circadian rhythm and antiepileptic drugs is recommended. The HRV is also under the effect of circadian variation in its nature. The alternation of HRV around the ictal period is directly due to seizures, but there may be an underlying effect of the circadian variation. Likewise, even though the included patients stopped taking antiepileptic drugs during their hospitalization, there could be long-lasting effects of previously taken antiepileptic drugs on ANS, which may cause alternations in HRV. Therefore, to clarify those underlying effects on HRV during seizures, further studies are recommended to be performed with cohorts monitored for a long term.

## 5. Conclusions

In this study, we investigated the differences between TLE and FLE patients with ultra-short-term HRV analysis. We showed that the patients with TLE and FLE had different HRV profiles in the pre-ictal, ictal and post-ictal intervals. The TLE patients exhibited elevated sympathetic or vagal activity during the pre-ictal condition, while the FLE patients showed a marked increment and decrement in sympathetic tone when entering and leaving the ictal period. The TLE patients showed sustained post-ictal elevation in sympathetic and vagal activity. These results could be useful in two ways. First, the acute alteration of cardiac functions during seizures could give information on lateralization of the seizure, since the central autonomic control centers could be hemispherically localized. Secondly, these findings could be used to understand the underlying pathophysiological mechanisms of SUDEP. The exact mechanism of SUDEP is still unknown and multifactorial, but cardiovascular dysfunction could be one of the triggers. Therefore, the ultra-short-term HRV analysis, which is useful to monitor the acute sympathetic or vagal activity around the ictal, could predict the risk of fatal cardiovascular malfunctions in the future.

## Figures and Tables

**Table 1:** Demographical and clinical information of patients in the study population.

	Total (n: 58)	FLE Group (n: 12)	TLE Group (n: 46)	*p*-Value *
Sex (M/F)	36/22	9/3	27/19	0.22
Age at evaluation	33.1 ± 15.7	18.3 ± 7.8	36.5 ± 14.4	0.01
Age at seizure onset	20.3 ± 16.6	16.3 ± 16.0	21.3 ± 16.6	0.29
Disease duration (years)	15.8 ± 9.7	11.5 ± 5.6	16.9 ± 10.4	0.07
Etiology of epilepsy				
Cryptogenic	11	8	3	
Hippocampal sclerosis	26	0	26	
Cortical malformation	3	2	1	
Tumor	4	1	3	
Vascular malformation	5	1	4	
History of encephalitis	5	0	5	
Destructive lesion	1	0	1	
Frequency of seizure				
Daily	13	5	8	
Weekly	22	5	17	
Monthly	21	2	19	
Yearly	2	0	2	
Number of seizures	5.3 ± 3.3	5.8 ± 3.4	5.2 ± 3.2	0.57
Duration of seizures (sec)	94.4 ± 53.2	79.4 ± 59.9	98.8 ± 50.4	0.02
Number of AEDs previously used		2.3 ± 1.4	2.6 ± 1.2	0.28
Laterality (Rt/Lt/bilateral)				
Rt		6	19	
Lt		4	21	
Bilateral or non-lateralized		2	6	

FLE: frontal lobe epilepsy; TLE: temporal lobe epilepsy; AED: antiepileptic drug; * *p*-value indicates significant difference between epilepsy types (FLE and TLE).

**Table 2:** Independent *t*-test of the HRV parameters between FLE and TLE in each interval.

Interval	HRV Parameter	FLE Group	TLE Group	*p*-Value
Pre-ictal	Mean RRi (ms)	**865.7 ± 166.3**	**811.4 ± 156.5**	**0.012**
	SDNN (ms)	73.1 ± 44.6	79.3 ± 42.5	0.296
	RMSSD (ms)	**45.2 ± 25.9**	**54.4 ± 32.3**	**0.031**
	Normalized LF (%)	**26.7 ± 11.2**	**30.7 ± 13.5**	**0.023**
	Normalized HF (%)	21.0 ± 14.4	24.7 ± 16.2	0.088
	LF/HF	2.0 ± 1.7	2.1 ± 2.3	0.892
Ictal	Mean RRi (ms)	**632.6 ± 129.0**	**559.7 ± 97.5**	**<0.001**
	SDNN (ms)	98.5 ± 44.0	96.4 ± 46.1	0.734
	RMSSD (ms)	56.1 ± 34.2	60.1 ± 39.8	0.443
	Normalized LF (%)	**37.2 ± 21.0**	**27.3 ± 20.3**	**<0.001**
	Normalized HF (%)	16.9 ± 15.3	20.0 ± 18.5	0.190
	LF/HF	**4.8 ± 5.0**	**3.1 ± 4.8**	**0.010**
Post-ictal	Mean RRi (ms)	**719.2 ± 149.2**	**657.8 ± 129.4**	**<0.001**
	SDNN (ms)	85.0 ± 41.9	85.8 ± 49.6	0.897
	RMSSD (ms)	43.4 ± 23.4	50.5 ± 32.3	0.088
	Normalized LF (%)	25.8 ± 13.1	27.8 ± 14.7	0.308
	Normalized HF (%)	17.0 ± 13.9	17.2 ± 12.0	0.901
	LF/HF	2.7 ± 2.4	2.5 ± 2.5	0.726

Mean RRi: mean number of inter-beat intervals between successive heartbeats; SDNN: a standard deviation of the normal-to-normal interval; RMSSD: square root of the mean of the sum of the squares of the differences between consecutive normal-to-normal intervals; normalized LF: the relative power of the frequency band (0.04–0.15 Hz); normalized HF: the relative power of the high-frequency band (0.15–0.4 Hz); LF/HF: a ratio of low- to high-frequency power. Bold indicates a *p*-value less than 0.05.

**Table 3:** Repeated measures ANOVA for HRV parameters.

HRV Parameter	Source	$\eta_{p}^{2}$	F	*p*-Value
Mean RRi (ms)	Type	**0.037**	**35.49**	**<0.001**
	Interval	**0.280**	**179.07**	**<0.001**
	TYPE × Interval	**0.001**	**0.26**	0.767
SDNN (ms)	Type	0.000	0.20	0.651
	Interval	**0.025**	**11.89**	**<0.001**
	TYPE × Interval	0.001	0.45	0.636
RMSSD (ms)	Type	**0.007**	**6.58**	**0.011**
	Interval	**0.138**	**6.46**	**0.002**
	TYPE × Interval	0.007	0.32	0.729
Normalized LF (%)	Type	0.001	1.02	0.313
	Interval	**0.013**	**6.23**	**0.002**
	TYPE × Interval	**0.025**	**11.55**	**<0.001**
Normalized HF (%)	Type	0.004	3.75	0.053
	Interval	**0.018**	**8.21**	**<0.001**
	TYPE × Interval	0.002	0.79	0.454
LF/HF	Type	**0.005**	**5.00**	**0.026**
	Interval	**0.036**	**17.21**	**<0.001**
	TYPE × Interval	**0.009**	**4.39**	**0.013**

$\eta_{p}^{2}$: effect size as partial eta squared; Mean RRi: mean number of inter-beat intervals between successive heartbeats; SDNN: a standard deviation of the normal-to-normal interval; RMSSD: square root of the mean of the sum of the squares of the differences between consecutive normal-to-normal intervals; normalized LF: the relative power of the frequency band (0.04–0.15 Hz); normalized HF: the relative power of the high-frequency band (0.15–0.4 Hz); LF/HF: a ratio of low- to high-frequency power. Bold indicates a *p*-value less than 0.05.

**Table 4:** Multiple linear regression analysis with HRV parameters.

Independent Variables	β	t	*p*-Value
With the difference in the normalized LF between pre-ictal and ictal HRV as the dependent variables			
Sex	3.39	1.13	0.258
Number of AED	−2.15	−1.92	0.056
Epilepsy type	9.36	2.59	**0.010**
Seizure frequency	−5.75	−3.24	**0.001**
R^2^ = 0.101; adjusted R^2^ = 0.090			
With the difference in the normalized LF between ictal and post-ictal HRV as the dependent variables			
Sex	−1.60	−0.49	0.622
Number of AED	1.08	0.89	0.374
Epilepsy type	−6.76	−1.73	0.084
Seizure frequency	6.25	−3.26	**0.001**
R^2^ = 0.072; adjusted R^2^ = 0.060			

β: standardized regression coefficient; HRV: heart rate variability; normalized LF: the relative power of the frequency band (0.04–0.15 Hz). Bold indicates a *p*-value less than 0.05.

## Data Availability

The data are not publicly available due to ethical restrictions.

## Supplementary Materials

The following are available online at https://www.mdpi.com/article/10.3390/medicina57070666/s1. Table S1: Post hoc Tukey–Kramer multiple comparison tests for HRV parameters between FLE and TLE groups.

## References

[1] Fiest K.M., Sauro K.M., Wiebe S., Patten S.B., Kwon C.S., Dykeman J., Pringsheim T., Lorenzetti D.L., Jetté N. (2017). Prevalence and incidence of epilepsy. Neurology.

[2] Murray C.J.L., Vos T., Lozano R., Naghavi M., Flaxman A.D., Michaud C., Ezzati M., Shibuya K., Salomon J.A., Abdalla S. (2012). Disability-adjusted life years (DALYs) for 291 diseases and injuries in 21 regions, 1990–2010: A systematic analysis for the Global Burden of Disease Study 2010. Lancet.

[3] Commission on Classification and Terminology of the International League Against Epilepsy (1981). Proposal for Revised Clinical and Electroencephalographic Classification of Epileptic Seizures: From the Commission on Classification and Terminology of the International League against Epilepsy. Epilepsia.

[4] O’Brien T.J., Mosewich R.K., Britton J.W., Cascino G.D., So E.L. (2008). History and seizure semiology in distinguishing frontal lobe seizures and temporal lobe seizures. Epilepsy Res..

[5] Fisher R.S., Cross J.H., French J.A., Higurashi N., Hirsch E., Jansen F.E., Lagae L., Moshé S.L., Peltola J., Roulet Perez E. (2017). Operational classification of seizure types by the International League Against Epilepsy: Position Paper of the ILAE Commission for Classification and Terminology. Epilepsia.

[6] Foldvary-Schaefer N., Unnwongse K. (2011). Localizing and lateralizing features of auras and seizures. Epilepsy Behav..

[7] Ansakorpi H., Korpelainen J.T., Suominen K., Tolonen U., Myllylä V.V., Isojärvi J.I.T. (2000). Interictal cardiovascular autonomic responses in patients with temporal lobe epilepsy. Epilepsia.

[8] Druschky A. (2001). Interictal cardiac autonomic dysfunction in temporal lobe epilepsy demonstrated by [123I]metaiodobenzylguanidine-SPECT. Brain.

[9] Sathyaprabha T.N., Satishchandra P., Netravathi K., Sinha S., Thennarasu K., Raju T.R. (2006). Cardiac autonomic dysfunctions in chronic refractory epilepsy. Epilepsy Res..

[10] Harnod T., Yang C.C.H., Hsin Y.L., Wang P.J., Shieh K.R., Kuo T.B.J. (2009). Heart rate variability in patients with frontal lobe epilepsy. Seizure.

[11] Ravindran K., Powell K.L., Todaro M., O’Brien T.J. (2016). The pathophysiology of cardiac dysfunction in epilepsy. Epilepsy Res..

[12] Mukherjee S., Tripathi M., Chandra P.S., Yadav R., Choudhary N., Sagar R., Bhore R., Pandey R.M., Deepak K.K. (2009). Cardiovascular autonomic functions in well-controlled and intractable partial epilepsies. Epilepsy Res..

[13] Moseley B.D., Wirrell E.C., Nickels K., Johnson J.N., Ackerman M.J., Britton J. (2011). Electrocardiographic and oximetric changes during partial complex and generalized seizures. Epilepsy Res..

[14] Rocamora R., Kurthen M., Lickfett L., Von Oertzen J., Elger C.E. (2003). Cardiac asystole in epilepsy: Clinical and neurophysiologic features. Epilepsia.

[15] Sevcencu C., Struijk J.J. (2010). Autonomic alterations and cardiac changes in epilepsy. Epilepsia.

[16] Barot N., Nei M. (2019). Autonomic aspects of sudden unexpected death in epilepsy (SUDEP). Clin. Auton. Res..

[17] Eggleston K.S., Olin B.D., Fisher R.S. (2014). Ictal tachycardia: The head-heart connection. Seizure.

[18] Tinuper P., Bisulli F., Cerullo A., Carcangiu R., Marini C., Pierangeli G., Cortelli P. (2001). Ictal bradycardia in partial epileptic seizures: Autonomic investigation in three cases and literature review. Brain.

[19] Schuele S.U., Bermeo A.C., Alexopoulos A.V., Locatelli E.R., Burgess R.C., Dinner D.S., Foldvary-Schaefer N. (2007). Video-electrographic and clinical features in patients with ictal asystole. Neurology.

[20] Dünser M.W., Hasibeder W.R. (2009). Sympathetic overstimulation during critical illness: Adverse effects of adrenergic stress. J. Intensive Care Med..

[21] Suna N., Suna I., Gutmane E., Kande L., Karelis G., Viksna L., Folkmanis V. (2021). Electrocardiographic Abnormalities and Mortality in Epilepsy Patients. Medicina.

[22] Bateman L.M., Li C.S., Seyal M. (2008). Ictal hypoxemia in localization-related epilepsy: Analysis of incidence, severity and risk factors. Brain.

[23] Devinsky O. (2011). Sudden, unexpected death in epilepsy. N. Engl. J. Med..

[24] Malik M., Camm A.J., Bigger J.T., Breithardt G., Cerutti S., Cohen R.J., Coumel P., Fallen E.L., Kennedy H.L., Kleiger R.E. (1996). Heart rate variability. Standards of measurement, physiological interpretation, and clinical use. Eur. Heart J..

[25] Myers K.A., Sivathamboo S., Perucca P. (2018). Heart rate variability measurement in epilepsy: How can we move from research to clinical practice?. Epilepsia.

[26] Monté C.P.J.A., Arends J.B.A.M., Tan I.Y., Aldenkamp A.P., Limburg M., de Krom M.C.T.F.M. (2007). Sudden unexpected death in epilepsy patients: Risk factors. A systematic review. Seizure.

[27] Persson H., Kumlien E., Ericson M., Tomson T. (2005). Preoperative heart rate variability in relation to surgery outcome in refractory epilepsy. Neurology.

[28] Son W.H., Hwang W.S., Koo D.L., Hwang K.J., Kim D.Y., Seo J.-H., Na G.-Y., Joo E.Y., Hong S.B., Seo D.-W. (2016). The Difference in Heart Rate Change between Temporal and Frontal Lobe Seizures during Peri-ictal Period. J. Epilepsy Res..

[29] Shaffer F., McCraty R., Zerr C.L. (2014). A healthy heart is not a metronome: An integrative review of the heart’s anatomy and heart rate variability. Front. Psychol..

[30] Malik M. (1996). Heart rate variability: Standards of measurement, physiological interpretation, and clinical use. Circulation.

[31] Kleiger R.E., Miller J.P., Bigger J.T., Moss A.J. (1987). Decreased heart rate variability and its association with increased mortality after acute myocardial infarction. Am. J. Cardiol..

[32] Nolan J., Batin P.D., Andrews R., Lindsay S.J., Brooksby P., Mullen M., Baig W., Flapan A.D., Cowley A., Prescott R.J. (1998). Prospective study of heart rate variability and mortality in chronic heart failure: Results of the United Kingdom heart failure evaluation and assessment of risk trial (UK-Heart). Circulation.

[33] Berkoff D.J., Cairns C.B., Sanchez L.D., Moorman C.T. (2007). Heart rate variability in elite american track-and-field athletes. J. Strength Cond. Res..

[34] Abhishekh H.A., Nisarga P., Kisan R., Meghana A., Chandran S., Raju T., Sathyaprabha T.N. (2013). N. Influence of age and gender on autonomic regulation of heart. J. Clin. Monit. Comput..

[35] Seppälä S., Laitinen T., Tarvainen M.P., Tompuri T., Veijalainen A., Savonen K., Lakka T. (2014). Normal values for heart rate variability parameters in children 6-8 years of age: The PANIC Study. Clin. Physiol. Funct. Imaging.

[36] Shaffer F., Ginsberg J.P. (2017). An Overview of Heart Rate Variability Metrics and Norms. Front. Public Heal..

[37] Pernice R., Faes L., Kotiuchyi I., Stivala S., Busacca A., Popov A., Kharytonov V. (2019). Time, frequency and information domain analysis of short-term heart rate variability before and after focal and generalized seizures in epileptic children. Physiol. Meas..

[38] Stavrinou M.L., Sakellaropoulos G.C., Trachani E., Sirrou V., Polychronopoulos P., Nikiforidis G., Chroni E. (2014). Methodological issues in the spectral analysis of the heart rate variability: Application in patients with epilepsy. Biomed. Signal Process. Control.

[39] Evrengül H., Tanriverdi H., Dursunoglu D., Kaftan A., Kuru O., Unlu U., Kilic M. (2005). Time and frequency domain analyses of heart rate variability in patients with epilepsy. Epilepsy Res..

[40] Yang Z., Liu H., Meng F., Guan Y., Zhao M., Qu W., Hao H., Luan G., Zhang J., Li L. (2018). The analysis of circadian rhythm of heart rate variability in patients with drug-resistant epilepsy. Epilepsy Res..

[41] Salahuddin L., Cho J., Jeong M.G., Kim D. Ultra short term analysis of heart rate variability for monitoring mental stress in mobile settings. Proceedings of the Annual International Conference of the IEEE Engineering in Medicine and Biology Proceedings.

[42] Nussinovitch U., Elishkevitz K.P., Katz K., Nussinovitch M., Segev S., Volovitz B., Nussinovitch N. (2011). Reliability of ultra-short ECG indices for heart rate variability. Ann. Noninvasive Electrocardiol..

[43] Nussinovitch U., Cohen O., Kaminer K., Ilani J., Nussinovitch N. (2012). Evaluating reliability of ultra-short ECG indices of heart rate variability in diabetes mellitus patients. J. Diabetes Complicat..

[44] Shaffer F., Shearman S., Meehan Z.M. (2016). The Promise of Ultra-Short-Term (UST) Heart Rate Variability Measurements. Biofeedback.

[45] Shaffer F., Meehan Z.M., Zerr C.L. (2020). A Critical Review of Ultra-Short-Term Heart Rate Variability Norms Research. Front. Neurosci..

[46] Munoz M.L., Van Roon A., Riese H., Thio C., Oostenbroek E., Westrik I., De Geus E.J.C., Gansevoort R., Lefrandt J., Nolte I.M. (2015). Validity of (Ultra-)Short recordings for heart rate variability measurements. PLoS ONE.

[47] Zhao Z.D., Chen Y.Q. A new method for removal of baseline wander and power line interference in ECG signals. Proceedings of the 2006 International Conference on Machine Learning and Cybernetics.

[48] Elgendi M., Jonkman M., Deboer F. Frequency bands effects on QRS detection. Proceedings of the 3rd International Conference on Bio-inpsired Systems and Signal Processing—BIOSIGNALS 2010.

[49] Kadambe S., Murray R., Paye Boudreaux-Bartels G. (1999). Wavelet transform-based QRS complex detector. IEEE Trans. Biomed. Eng..

[50] Nabil D., Bereksi Reguig F. (2015). Ectopic beats detection and correction methods: A review. Biomed. Signal Process. Control.

[51] Ruf T. (1999). The Lomb-Scargle periodogram in biological rhythm research: Analysis of incomplete and unequally spaced time-series. Biol. Rhythm Res..

[52] Clifford G.D., Tarassenko L. (2005). Quantifying errors in spectral estimates of HRV due to beat replacement and resampling. IEEE Trans. Biomed. Eng..

[53] Fonseca D.S., Netto A.D.A., Ferreira R.B., De Sa A.M.F.L.M. Lomb-scargle periodogram applied to heart rate variability study. Proceedings of the ISSNIP Biosignals and Biorobotics Conference, BRC.

[54] Reyes del Paso G.A., Langewitz W., Mulder L.J.M., van Roon A., Duschek S. (2013). The utility of low frequency heart rate variability as an index of sympathetic cardiac tone: A review with emphasis on a reanalysis of previous studies. Psychophysiology.

[55] Hallioglu O., Okuyaz C., Mert E., Makharoblidze K. (2008). Effects of antiepileptic drug therapy on heart rate variability in children with epilepsy. Epilepsy Res..

[56] Stein P.K., Bosner M.S., Kleiger R.E., Conger B.M. (1994). Heart rate variability: A measure of cardiac autonomic tone. Am. Heart J..

[57] Sztajzel J. (2004). Heart rate variability: A noninvasive electrocardiographic method to measure the autonomic nervous system. Swiss Med. Wkly..

[58] Hayter A.J. (1984). A Proof of the Conjecture that the Tukey-Kramer Multiple Comparisons Procedure is Conservative. Ann. Stat..

[59] Umetani K., Singer D.H., McCraty R., Atkinson M. (1998). Twenty-four hour time domain heart rate variability and heart rate: Relations to age and gender over nine decades. J. Am. Coll. Cardiol..

[60] Jansen K., Lagae L. (2010). Cardiac changes in epilepsy. Seizure.

[61] Nei M., Ho R.T., Abou-Khalil B.W., Drislane F.W., Liporace J., Romeo A., Sperling M.R. (2004). EEG and ECG in Sudden Unexplained Death in Epilepsy. Epilepsia.

[62] Rugg-Gunn F.J., Simister R.J., Squirrell M., Holdright D.R., Duncan P.J.S. (2004). Cardiac arrhythmias in focal epilepsy: A prospective long-term study. Lancet.

[63] Leutmezer F., Schernthaner C., Lurger S., Pötzelberger K., Baumgartner C. (2003). Electrocardiographic changes at the onset of epileptic seizures. Epilepsia.

[64] Schernthaner C., Lindinger G., Pötzelberger K., Zeiler K., Baumgartner C. (1999). Autonomic epilepsy—The influence of epileptic discharges on heart rate and rhythm. Wien Klin. Wochenschr..

[65] Beissner F., Meissner K., Bär K.J., Napadow V. (2013). The autonomic brain: An activation likelihood estimation meta-analysis for central processing of autonomic function. J. Neurosci..

[66] Kanbara K., Fukunaga M. (2016). Links among emotional awareness, somatic awareness and autonomic homeostatic processing. Biopsychosoc. Med..

[67] Devinsky O., Hesdorffer D.C., Thurman D.J., Lhatoo S., Richerson G. (2016). Sudden unexpected death in epilepsy: Epidemiology, mechanisms, and prevention. Lancet Neurol..

[68] Lotufo P.A., Valiengo L., Benseñor I.M., Brunoni A.R. (2012). A systematic review and meta-analysis of heart rate variability in epilepsy and antiepileptic drugs. Epilepsia.

[69] Shen H.Y., Li T., Boison D. (2010). A novel mouse model for sudden unexpected death in epilepsy (SUDEP): Role of impaired adenosine clearance. Epilepsia.

