# Supplementary material for: Comparing Ictal Cardiac Autonomic Changes in Patients with Frontal Lobe Epilepsy and Temporal Lobe Epilepsy by Ultra-Short-Term Heart Rate Variability Analysis
Source: Medicina (Kaunas). 2021 Jun 28;57(7):666. doi: 10.3390/medicina57070666 (PMC8304923; doi:10.3390/medicina57070666)
Supplement: Supplementary file 1 [file medicina-57-00666-s001.zip › medicina-1266743-supplementary.pdf]

Table S1. Post-hoc Tukey-Kramer multiple comparison tests for HRV parameters between FLE and TLE groups.

HRV parameter	Type	Pairs	Mean difference	TK HSD	Significance
mean RRi	FLE group	**Pre–ictal**	**233.0795**	**14.495**	*
		**Pre-post**	**146.5400**	**9.113**	*
		**Ictal–post**	**86.5395**	**5.3817**	*
	TLE group	**Pre–ictal**	**251.7075**	**28.9238**	*
		**Pre-post**	**153.5154**	**17.6405**	*
		**Ictal–post**	**98.1921**	**11.2833**	*
SDNN	FLE group	**Pre–ictal**	**25.4096**	**4.6636**	*
		Pre–post	11.8412	2.1733	n.s
		Ictal–post	13.5684	2.4903	n.s
	TLE group	**Pre–ictal**	**17.1837**	**5.8276**	*
		Pre–post	6.5678	2.2274	n.s
		Ictal–post	10.6159	3.6002	n.s
RMSSD	FLE group	Pre–ictal	10.8694	2.7085	n.s
		Pre–post	1.8342	0.4571	n.s
		Ictal–post	12.7036	3.1656	n.s
	TLE group	Pre–ictal	5.7766	2.6598	n.s
		Pre–post	3.8408	1.7685	n.s
		**Ictal–post**	**9.6174**	**4.4283**	*
normalized LF	FLE group	**Pre–ictal**	**10.482**	**5.392**	*
		Pre–post	0.897	0.462	n.s
		**Ictal–post**	**11.379**	**5.853**	*
	TLE group	Pre–ictal	3.423	3.253	n.s
		Pre–post	2.923	2.778	n.s
		Ictal–post	0.500	0.475	n.s
normalized HF	FLE group	Pre–ictal	4.1903	2.2561	n.s
		Pre–post	4.0467	2.1788	n.s
		Ictal–post	0.1436	0.0773	n.s
	TLE group	**Pre–ictal**	**4.6807**	**4.6567**	*
		**Pre-post**	**7.5185**	**7.4798**	*
		Ictal–post	2.8377	2.8232	n.s
LF/HF ratio	FLE group	**Pre–ictal**	**2.720**	**6.744**	*
		Pre–post	0.624	1.548	n.s
		**Ictal–post**	**2.096**	**5.196**	*
	TLE group	**Pre–ictal**	**0.983**	**4.501**	*
		Pre–post	0.466	2.133	n.s
		Ictal–post	0.517	2.368	n.s

The critical q-value for an alpha level of 0.05 was 4.0385. Mean RRi, mean of inter-beat intervals between successive heartbeats; SDNN, a standard deviation of the normal-to-normal interval; RMSSD, square root of the mean of the sum of the squares of differences between consecutive normal-to-normal intervals; normalized LF, the relative power of the frequency band (0.04-0.15 Hz); normalized HF, the relative power of the high-frequency band (0.15-0.4 Hz); LF/HF, a ratio of low- to high-frequency power; * Bold indicates p-value less than 0.05.
